# Persistent exercise limitation after successful pulmonary endoarterectomy: frequency and determinants

**DOI:** 10.1186/s12931-019-1002-5

**Published:** 2019-02-14

**Authors:** Angelo G. Corsico, Andrea M. D’Armini, Valentina Conio, Antonio Sciortino, Maurizio Pin, Valentina Grazioli, Giulia Di Vincenzo, Rita Di Domenica, Anna Celentano, Benedetta Vanini, Amelia Grosso, Erica Gini, Federica Albicini, Vera N. Merli, Vanessa Ronzoni, Stefano Ghio, Catherine Klersy, Isa Cerveri

**Affiliations:** 1 Division of Respiratory Diseases, IRCCS Policlinico San Matteo Foundation, Viale Golgi 19, 27100 Pavia, Italy; 2 Department of Internal Medicine and Therapeutics, University of Pavia, Pavia, Italy; 3 Department of Cardio-Thoracic and Vascular Surgery, IRCCS Policlinico San Matteo Foundation, Pavia, Italy; 4 Department of Clinical-Surgical, Diagnostic and Pediatric Sciences, University of Pavia, Pavia, Italy; 5 Division of Cardiology, IRCCS Policlinico San Matteo Foundation, Pavia, Italy; 6 Service of Biometrics and Statistics, IRCCS Policlinico San Matteo Foundation, Pavia, Italy

**Keywords:** Hypertension, pulmonary, Embolism, Surgical procedures, Physical exertion

## Abstract

**Background:**

After successful pulmonary endoarterectomy (PEA), patients may still suffer from exercise limitation, despite normal pulmonary vascular resistance. We sought to assess the proportion of these patients after the extension of PEA to frail patients, and the determinants of exercise limitation.

**Methods:**

Out of 553 patients treated with PEA from 2008 to 2016 at our institution, a cohort of 261 patients was followed up at 12 months. They underwent clinical, haemodynamic, echocardiographic, respiratory function tests and treadmill exercise testing. A reduced exercise capacity was defined as Bruce test distance < 400 m.

**Results:**

Eighty patients did not had exercise testing because of inability to walk on treadmill and/or ECG abnormalities Exercise limitation 12 months after PEA was present in 74/181 patients (41, 95%CI 34 to 48%). The presence of COPD was more than double in patients with exercise limitation than in the others. Patients with persistent exercise limitation had significantly higher mPAP, PVR, HR and significantly lower RVEF, PCa, CI, VC, TLC, FEV_1_, FEV_1_/VC, D_LCO_, HbSaO_2_ than patients without. The multivariable model shows that PCa at rest and TAPSE are important predictors of exercise capacity. Age, COPD, respiratory function parameters and unilateral surgery were also retained.

**Conclusions:**

After successful PEA, most of the patients recovered good exercise tolerance. However, about 40% continues to suffer from limitation to a moderate intensity exercise. Besides parameters of right ventricular function, useful information are provided by respiratory function parameters and COPD diagnosis. This could be useful to better address the appropriate therapeutic approach.

**Electronic supplementary material:**

The online version of this article (10.1186/s12931-019-1002-5) contains supplementary material, which is available to authorized users.

## Background

Pulmonary endoarterectomy (PEA) is the treatment of choice for chronic thromboembolic pulmonary hypertension (CTEPH) [1, 2]. Several recent reports demonstrated favorable effects on long-term survival and cardiopulmonary function recovery [3–5]. As a results of the growing experience worldwide, the evolving surgical techniques and the increased number of patients who could benefit from excellent surgical outcome, the patients’ age at surgery and the severity of preoperative pulmonary hypertension have significantly increased [6, 7]. Consequently, many operated patients can present comorbidities and in particular, a number of them can suffer also from chronic respiratory diseases [7, 8].

It is recognized that after PEA, successfully operated patients can continue to suffer from a limitation of exercise capacity [9]. Recently, many studies aimed to explore the cardiopulmonary exercise test (CPET) profile and the pulmonary response to exercise. All of the studies concluded that exercise was impaired by a cardiac rather than ventilator limitation [1, 9–11]. Most of the previous series used sophisticated and complex protocols specifically addressed to study the pathophysiological mechanisms by CPET and consequently included a very small number of patients. This precluded the evaluation of the proportion of patients with exercise limitation after PEA. On the other hand, the simple 6-min walking test has gained popularity, albeit it provides different information, being less demanding that CPET and imprecise in answering important clinical questions [12].

Taking into account the enlargement of the number of patients who can now access the intervention, we hypothesized that age, comorbidity, lung function and COPD can play a role in the persistent exercise limitation after PEA. The aim of our study was to define the proportion of patients who still suffer from exercise limitation and to analyze its determinants, with particular attention to age and clinical and functional respiratory abnormalities at rest, in patients capable of doing exercise at 12 month after PEA.

## Methods

### Patients

**Fig. 1:**
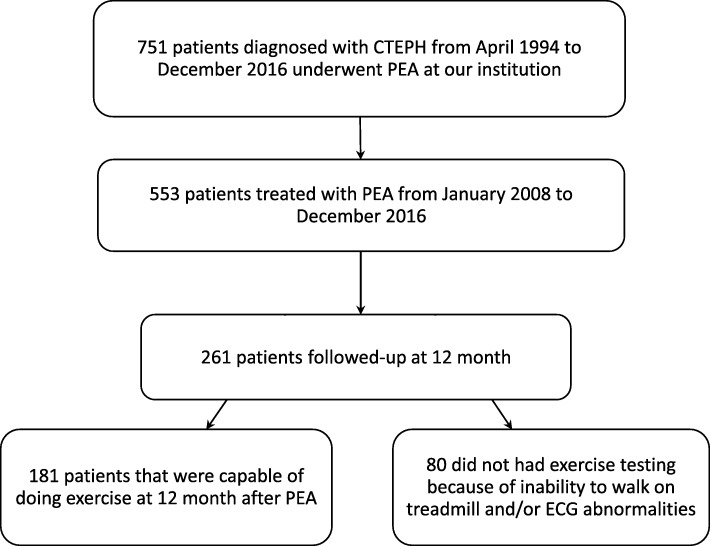
Diagram for flow of study participants

From April 1994 to December 2016, 751 patients diagnosed with CTEPH, according to published standardized protocol underwent PEA at our institution [3, 11]. The anaesthesiological and surgical methods used have been extensively reported and discussed elsewhere [6]. Patients underwent clinical (with New York Heart Association (NYHA) class), haemodynamic, echocardiographic and respiratory function evaluations preoperatively and after surgery at hospital discharge, at 3 months and then every year for the following 5 years. Out of the 553 patients treated with PEA from January 2008 to December 2016, a cohort of 261 patients were followed-up at 12 months. The present study is a retrospective evaluation of the 181 patients that were capable of doing exercise at 12 month after PEA (80 did not had exercise testing because of inability to walk on treadmill and/or electrocardiogram (ECG) abnormalities). The flow of subjects is shown in Fig. 1.

PEA was a bilateral procedure in 163 (90%) and unilateral in 18 (10%) patients. Unilateral procedure was chosen for frail patients with bilateral disease or for patients with controlateral lung parenchymal disease or with technical difficulties (redo, pericarditis).

### Study protocol

Right heart catheterization was performed through the right internal jugular vein using a flow-directed, balloon-tipped Swan–Ganz catheter (7.5F; Edwards Lifesciences, Irvine, CA, USA). The following parameters were measured or calculated: heart rate (HR), cardiac output (CO), cardiac index (CI), right ventricular ejection fraction (RVEF), pulmonary artery pressure (mPAP), capillary wedge pressure, pulmonary vascular resistance (PVR) and pulmonary arterial compliance (PCa) (calculated as stroke volume/pulse pressure).

A standard M-mode, two-dimensional and Doppler echocardiographic study was performed using commercially available equipment. The examination included the tricuspid annular plane systolic excursion (TAPSE).

The lung function evaluation included spirometry, lung volumes, single breath carbon monoxide diffusing capacity (D_L_co) and pulse oximetry arterial oxygen saturation (HbSaO_2_).

The clinical evaluation included the assessment of comorbidities and chronic respiratory symptoms. COPD diagnosis was confirmed with spirometry finding of forced expiratory volume in 1 s (FEV_1_) to vital capacity (VC) ratio < lower limit of normal (LLN).

The treadmill exercise without the measurement of respiratory gas exchange was performed according to the modified Bruce protocol, the distance (in meters) walked was measured to describe functional capacity [3].

All patients signed an informed consent agreement, approved by the institutional review board of Fondazione IRCCS Policlinico San Matteo (Pavia, Italy) for longitudinal, nonpharmacological, non-sponsored studies, which complies with the Italian legislation (Codex on Privacy, D. Lgs. 30 June 2003, n. 196).

### Statistical analysis

Data were described as mean ± standard deviation (sd) or median (25th–75th percentiles) if continuous and as counts (%) if categorical. A reduced exercise capacity was defined as Bruce protocol distance < 400 m. The prevalence of reduced exercise capacity and its 95% binomial exact confidence interval was computed. The association of patients’ characteristics with reduced exercise capacity was assessed by a logistic regression model. The unpaired Student t test and the Fisher exact test were used to compare functional variables between exercise capacity groups at 12 months. Non collinear variables with *p* < 0.2 in univariable analysis were included in a multivariable model. Model discrimination was assessed with the model area under the Receiver Operating Characteristic curve (LROC, the closer to 1, the better) and model calibration with the shrinkage coefficient.

Stata 14 (StataCorp LP, College Station, TX, USA) was used for computation, a two-sided *p*-value < 0.05 was considered statistically significant.

## Results

The characteristics of the 181 patients entered in the present study are reported in Table 1. Hemodynamic and respiratory variables 12 months after PEA are reported in Table 2. PEA was considered successful and none of the patients was taking specific medication for pulmonary hypertension.

**Table 1:** Patients’ characteristics

Subjects	181
Demographic data	
Age, *years,* mean ± sd (range)	61 ± 12 (21–84)
Male/Female, n.	81/100
BMI, *kg.m*^*−2*^, mean ± sd	27 ± 4
Smoking status	
Current smokers, n. (%)	13 (7)
Never smokers, n. (%)	94 (52)
Past smokers, n. (%)	74 (41)
Medical history	
NYHA Class I, n.	141
NYHA Class II-III, n.	40
Chronic cough and phlegm, n. (%)	32 (18)
Asthma, n. (%)	12 (7)
COPD, n. (%)	19 (10)

*BMI* Body Mass Index, *NYHA* New York Heart Association

**Table 2:** Hemodynamic and respiratory variables 12 months after PEA in the whole group and in those with or without reduced exercise capacity (defined as Bruce protocol distance < 400 m)

	Whole group	< 400 m	≥400 m	*p* value
mPAP, *mmHg*	23 ± 67	25 ± 8	21 ± 6	< 0.001
CI, *L/min/m*^*2*^	2.7 ± 0.5	2.6 ± 0.5	2.7 ± 0.5	0.05
RVEF, *%*	38 ± 8	36 ± 9	39 ± 7	< 0.05
PVR, dyne_*_ *s/cm*^*5*^	249 ± 135	301 ± 170	213 ± 89	< 0.0001
PCa, *mL/mmHg*	2.85 ± 1.09	2.37 ± 0.87	3.19 ± 1.09	< 0.0001
TAPSE, *mm*	15.9 ± 2.8	15.6 ± 2.8	16.1 ± 2.8	NS
VC, *L*	3.31 ± 1.00	2.84 ± 0.76	3.63 ± 1.01	< 0.0001
VC, *% pred.*	100 ± 19	99 ± 21	101 ± 17	NS
TLC, *L*	5.35 ± 1.35	4.92 ± 1.10	5.63 ± 1.43	< 0.001
TLC, *% pred.*	95 ± 15	94 ± 16	95 ± 14	NS
FEV_1_, *L*	2.37 ± 0.77	1.98 ± 0.59	2.63 ± 0.78	< 0.0001
FEV_1_, *% pred.*	92 ± 19	90 ± 22	94 ± 17	NS
FEV_1_/VC, *%*	72 ± 8	70 ± 8	73 ± 7	< 0.05
D_L_co_,_ *mL/min/mmHg*	5.55 ± 1.66	4.65 ± 1.32	6.14 ± 1.59	< 0.0001
D_L_co, *% pred.*	68 ± 15	63 ± 14	72 ± 14	< 0.001
HbSaO_2_, %	97 ± 2	96 ± 2	97 ± 1	< 0.0001
HR, *bpm*	84 ± 12	86 ± 12	82 ± 12	< 0.05

Data are presented as mean ± sd. *CI* cardiac index, *DLco* single breath carbon monoxide diffusing capacity, *FEV1* forced expiratory volume in 1 s, *HbSaO*_*2*_ pulse oximetry arterial oxygen saturation, *HR* heart rate, *mPAP* mean pulmonary arterial pressure, *PCa* pulmonary arterial compliance, *PVR* pulmonary vascular resistence, *RVEF* right ventricular ejection fraction, *TAPSE* tricuspid annular plane systolic excursion, *TLC* total lung capacity, *VC* vital capacity

Exercise limitation 12 months after PEA was present in 74 patients (41, 95%CI 34 to 48%). The distance covered during the Bruce test was 205 ± 114 m in those with reduced exercise capacity and 664 ± 170 m in the others. Older age (67 ± 10 vs 57 ± 12 years, *p* < 0.001), female sex (68% vs 47%, *p* < 0.01), NYHA class II-III (46% vs 6%, p < 0.001) and COPD (16% vs 7%, *p* < 0.05) were found to be associated with reduced exercise capacity. No association was found for body mass index, smoking history, chronic respiratory symptoms, and asthma.

Patients with exercise limitation 12 months after PEA had significantly higher mPAP, PVR, HR and significantly lower RVEF, PCa, CI, VC, TLC, FEV_1_, FEV_1_/VC, D_LCO_, HbSaO_2_ than patients without exercise limitation. The complete hemodynamic and lung function variable associations are reported in Table 2.

**Fig. 2:**
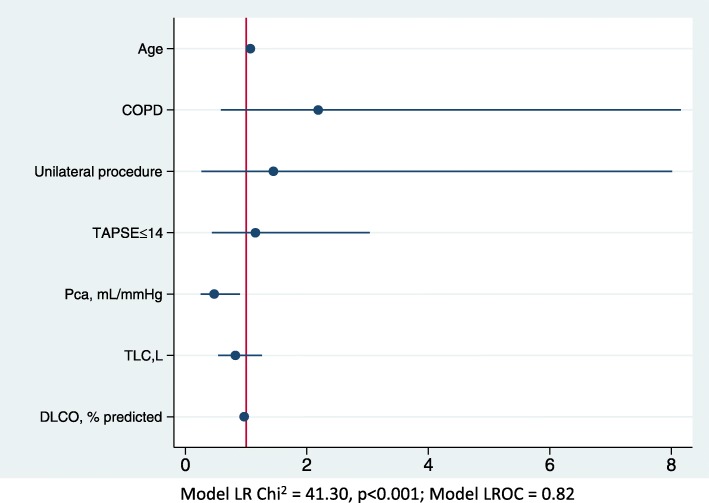
Forest plot of the multivariable logistic regression model for predicting reduced exercise capacity (definition based on distance walked) after pulmonary endarterectomy

A multivariable model including age, pulmonary arterial compliance, TAPSE, TLC, D_LCO_% predicted, COPD, and unilateral surgery procedure, showed a good discrimination (LROC = 0.82) and calibration (shrinkage coefficient = 0.83), and identified age and pulmonary arterial compliance as independent correlates of exercise limitation at 12 months (Fig. 2). A table in the additional file shows this in detail [see Additional file 1].

## Discussion

The present study shows that:

1. a persistent reduced exercise capacity is present in a substantial proportion of CTEPH patients who have undergone PEA (almost 40%) despite normalization of pulmonary vascular resistance;
2. this limitation is characterized by a multifactorial etiology involving also respiratory function abnormalities.

After successful PEA, most patients have a sustained improvement of functional status, hemodynamics, right ventricular function, and overall survival [3, 10, 13]. However, some recent studies have documented that in a number of patients exercise capacity does not completely return to normal, despite normalization of resting pulmonary artery pressures and pulmonary vascular resistance after PEA [9–11]. Abnormal increases in right ventricular afterload may be disclosed during exercise and may explain the reduced exercise capacity that persists in many patients [10]; another possible explanation is the extension of the indication for PEA to frail patients because of the improvement in the surgical procedures [7]. Unfortunately, these studies could not estimate the proportion of patients treated with PEA who continue to suffer from a persistent reduced exercise capacity because they included a very small number of patients or had a short follow-up. In order to overcome the limitations of previous studies we choose to use an incremental exercise test on treadmill according to the modified Bruce protocol. For the simplicity of execution for both patients and physicians, the Bruce test without the measurement of respiratory gas exchange was preferred to the formal CPET that is the gold standard. For most individuals, treadmill walking is a more familiar activity than cycling. Walking on the treadmill, however, is more complex than ordinary walking and this is a reason why not all the patients could perform the test. With respect to the 6 min walking test, the Bruce test is particularly useful in order to obtain a more reliable assessment of functional status. The 6MWT is a submaximal test unable to pick up all the improvement of the patients after PEA; it reflects better than other tests the activities of daily living but it is poorly demanding for our purposes.

In our study we could confirm that after PEA the majority of the patients recovered good exercise tolerance to a moderate intensity activity. However, despite normalization of pulmonary vascular resistance after PEA, almost 40% of the patients had a persistent reduced exercise capacity defined as the inability to cover at least 400 m at the Bruce exercise test. This is considered a moderate intensity activity, equivalent to 4 to 5 times the energy used by the body at rest, and is greater than ordinary physical activity in daily life [14]; in fact 80% of our patients were in class NYHA I. It should be considered that, on average, maximal oxygen uptake is 5–10% higher on a treadmill than on a cycle ergometer and the workload is more reproducible on treadmill than on a bike [15–17]. Our definition of exercise limitation as a walk distance < 400 m on a treadmill may seem somewhat arbitrarily; however, there are no standardized criteria to define what is exercise limitation in patients with CTEPH, particularly after PEA. Unfortunately, there are not predicted values for the distance covered during the Bruce test. A distance of 400 m at the Bruce test was chosen because it was the median walked distance covered at 1 year after PEA in our previous study [3].

The occurrence of persistent exercise limitation, documented in this study, increases the importance of the recent novel and significant findings on predictors. Some of these studies included both patients and controls who underwent cardiopulmonary exercise testing, followed by simultaneous invasive mPAP measurement during incremental supine cycling exercise [9, 10]. Exercise intolerance after PEA in these patients was explained by abnormal pulmonary vascular reserve and chronotropic incompetence during exercise. In our previous study, using the same prospective dedicated database since the PEA program was started, pulmonary arterial compliance was confirmed to be an important predictor of exercise capacity evaluated 3 months after PEA [11]. In the present study, we extended our evaluation at 12 months after PEA, time in which the clinical conditions were stable and the exercise performance reliable.

Since 2007–2008 we adopted the intermittent hypothermic circulatory arrest technique with moderate, instead of deep, hypothermia and we could safely perform PEA even in the elderly and the referring physicians were more prone to suggest a surgical evaluation also in these patients [6]. Because the prevalence of comorbidities, particularly COPD, is growing with aging, respiratory function abnormalities and presence of COPD were included in this updated database analysis. The occurrence of COPD was 10% in the whole sample and it was more than double in patients with exercise limitation after PEA than in the others. In the last 10 years, the group of Cambridge and our group have published papers on PEA in the elderly [7, 8]. With aging, the presence of COPD inevitably it is likely to increase in patients considered for PEA. In our previous paper on PEA in the elderly COPD was present in 9.7% of patients < 70 years and 17.0% of those > 70 years [7]. No difference was observed in terms of long term survival between the two groups of patients. Nevertheless, the hospital mortality rate of 9.1% among the elderly was almost twice that of the control group (5.1%), which reflects the greater global frailty of these patients, the higher prevalence of preoperative comorbidities, and the reduced vital organ functional reserve [7]. Our finding that COPD and ventilator impairment at rest is related to exercise intolerance reinforces the concern about elderly patients.

Although the mean FEV1/VC ratio may appear near normal in the exercise limitation group, we used the LLN to define COPD in order to avoid misclassification [18]. Confirming the importance of airflow obstruction, we found that FEV1/VC is an independent predictor of reduced exercise capacity. Among lung function parameters, D_L_co was the other independent predictor; it may be abnormal long before spirometry or volume abnormalities are present in several diseases. Low D_L_co can occur both in restrictive and obstructive diseases, particularly in the presence of emphysema or pulmonary vascular diseases [19]. Interestingly, in our study D_L_co was the only lung function parameter that was significantly associated with exercise limitation both when considered in absolute value or in percent predicted. At variance, the lung volume variables (namely VC, TLC and FEV_1_) were significant only when expressed in absolute value. This difference could be explained by the importance on exercise capacity of lung size, that is reduced in females and with aging, rather than by the deviation from the normal (corresponding to the percent predicted). It is not surprising because our definition of exercise limitation was based on the threshold of 400 m regardless of age, gender and height.

Our multivariate model confirms that pulmonary compliance at rest and TAPSE are important predictors of exercise capacity. Age, COPD, respiratory function parameters and the type of surgery were also retained by the multivariable model. Old age may be associated with skeletal muscle deconditioning and might blunt recovery. COPD was associated with the highest odds ratio. Although, unilateral PEA could be offered to frail patients and/or in difficult situations to lessen operative complications and to have a shorter duration of cardiopulmonary bypass, surgeons should take into account its effect on the outcome, for adequately informing the patients.

As recently stated by task force on CTEPH at the World Symposia on Pulmonary Hypertension, medical therapy has a supporting role to improve symptoms and haemodynamics in these patients [20]. Claessen et al. well documented that hemodynamics or right ventricular function after PEA during exercise are partially improved with sildenafil. According to the authors, this provides the rationale for a role of pulmonary vasodilators in further improving pulmonary vascular physiology and functional capacity and raises the question about its clinical indication [10]. In the same manner, it is important to establish, in each patient, whether COPD and/or lung function abnormalities post-PEA can contribute to explain the residual gap in exercise capacity that still persists in patients even after pulmonary vasodilators. An adequate management of COPD, in particular with long-acting inhaled bronchodilators, the mainstay of therapy for these patients can be effective in the improvement of exercise capacity after PEA.

### Strengths and limitations

This is a retrospective evaluation of a cohort of patients treated with PEA from 2008 to 2016 and followed up at 12 months. Because of barriers to participation, the 1-year follow-up visit was not always performed in our national referral center; we included all consecutive incident patients who had completed the follow up protocol at 12 months at our institution. Additionally, 80 patients had not exercise testing because of inability to walk on treadmill or ECG abnormalities. The database was not modified over time, and all variables were collected prospectively.

From the beginning of the PEA program in Pavia in 1994, almost 800 PEA were performed at Foundation IRCCS Policlinico San Matteo (Pavia, Italy). During this single-center experience there was an evolution of the surgical techniques for PEA and we observed a progressive decline in mortality [3, 6]. We decided not to consider patients undergoing operation before 2008, because only thereafter we adopted moderate, instead of deep, hypothermia and the same team with the same surgeon performed PEA. Thus, the series was more homogeneous in the surgical techniques, in the decision about the operable and inoperable lesions, and the learning curve of the team.

One strength and, at the same time, one limitation of our study is that it did not include CPET that is the gold standard. The Bruce test without measurement of respiratory gas exchange is not a substitute of the CPET. However, the first aim of our study was to define the proportion of patients who still suffer from exercise limitation at 12 month after PEA and our analysis is based on registry data prospectively collected during normal clinical practice in patients undergoing PEA at our Institution and not on a specific trial. From this perspective, it was important for us to arrange an incremental exercise test easy to use for both physicians and patients, even if elderly or frail. Other studies who adopted the formal CPET could not estimate the proportion of patients treated with PEA who continue to suffer from a persistent reduced exercise capacity because they included a very small number of patients or had a short follow-up, and the complexity of the protocol was one limiting factor. Unfortunately, the use of a simplified instrumental exercise test without the measurement of respiratory gas exchange has reduced our ability to detect the cause or the relative role of cardiovascular, ventilator impairment, or deconditioning. Because of this limitation, we considered in our analysis the measurement of individual organ system function at rest as possible determinants of exercise limitation.

## Conclusions

PEA is a well tolerated and effective treatment for CTEPH even in the elderly. However, after successful PEA a consistent proportion of CTEPH patients continues to suffer from exercise limitation despite normalization of pulmonary vascular resistance. In our study that included elderly patients, besides cardiac parameters, COPD –a comorbidity that is growing with aging–, a reduced respiratory functional reserve (namely DLco and TLC) and the unilateral procedure that is deserved to frail patients, are important factors involved in the exercise response. These findings have to be taken into account when the physician informs the patients about potential benefits and risks of the procedure and highlight the importance of an optimal therapeutic approach of the associate respiratory diseases.

## Additional file

Additional file 1: **Table S1.**The data are relative to the Fig. 2 and report the multivariable logistic regression model for predicting reduced exercise capacity (definition based on distance walked) after pulmonary endarterectomy. (DOCX 12 kb)

## Abbreviation

BMI: Body Mass Index
CI: Cardiac index
CPET: Cardiopulmonary exercise test
CTEPH: Chronic thromboembolic pulmonary hypertension
DLco: Single breath carbon monoxide diffusing capacity
ECG: Electrocardiogram
FEV1: Forced expiratory volume in 1 s
HbSaO2: Pulse oximetry arterial oxygen saturation
HR: Heart rate
LROC: Receiver Operating Characteristic curve
mPAP: Mean pulmonary arterial pressure
NYHA: New York Heart Association
PCa: Pulmonary arterial compliance
PEA: Pulmonary endoarterectomy
PVR: Pulmonary vascular resistence
RV: Residual volume
RVEF: Right ventricular ejection fraction
sd: Standard deviation
TAPSE: Tricuspid annular plane systolic excursion
TLC: Total lung capacity
VC: Vital capacity
