# Supplementary material for: Persistent exercise limitation after successful pulmonary endoarterectomy: frequency and determinants
Source: Respir Res. 2019 Feb 14;20:34. doi: 10.1186/s12931-019-1002-5 (PMC6376724; doi:10.1186/s12931-019-1002-5)
Supplement: Supplementary file 1 — Table S1.The data are relative to the Fig. 2 and report the multivariable logistic regression model for predicting reduced exercise capacity (definition based on distance walked) after pulmonary endarterectomy. (DOCX 12 kb) [file 12931_2019_1002_MOESM1_ESM.docx]

**Multivariable logistic regression model for predicting reduced exercise capacity (definition based on distance walked) after pulmonary endarterectomy**

| **Variables** | **odds ratio (95% CI)** | **p-value** |
| --- | --- | --- |
| **Age, year** | **1.07 (1.02-1.12)** | **0.002** |
| COPD | 2.19 (0.59-8.16) | 0.244 |
| Unilateral procedure | 1.42 (0.28-72.6) | 0.862 |
| TAPSE, <14 mm | 0.47 (0.25-3.04) | 0.773 |
| **PCa, mL/mmHg** | **0.43 (0.23-0.90)** | **0.022** |
| TLC, L | 0.82 (0.54-1.26) | 0.371 |
| D_L_co, % predicted | 0.97 (0.93-1.00) | 0.083 |
|  | Model LR Chi^2^=41.30, p<0.0001 |  |

CI = cardiac index; DLco = single breath carbon monoxide diffusing capacity; PCa = pulmonary arterial compliance; TAPSE = tricuspid annular plane systolic excursion; TLC = total lung capacity.
